# Diminished satellite cell fusion and S6K1 expression in myotubes derived from skeletal muscle of low birth weight neonatal pigs

**DOI:** 10.14814/phy2.13075

**Published:** 2017-02-09

**Authors:** Ying Chen, Haibo Zhu, Sydney R. McCauley, Lidan Zhao, Sally E. Johnson, Robert P. Rhoads, Samer W. El‐Kadi

**Affiliations:** ^1^ Department of Animal and Poultry SciencesVirginia TechBlacksburgVirginia24061

**Keywords:** IGF‐I, low birth weight, satellite cells, skeletal muscle growth

## Abstract

Low birth weight (LBWT) is consistently associated with impaired postnatal muscle growth in mammals. Satellite cell (SC)‐mediated myonuclear incorporation precedes protein accumulation in the early stages of postnatal muscle development and growth. The objective of this study was to investigate proliferation and differentiation of SCs and the regulation of protein synthesis signaling in response to insulin‐like growth factor (IGF)‐I stimulation in SC‐derived myotubes of LBWT neonatal pigs. SCs isolated from *Longissimus dorsi* muscle of LBWT and NBWT pigs (3‐d‐old*, n *=* *8) were cultured and induced to proliferate and differentiate to myotubes in vitro. On day 3 of differentiation, myotubes were fasted in serum‐free media for 3 h and treated with human recombinant R^3^‐insulin‐like growth factor‐I (rh IGF‐I) at 0, 25, and 50 ng × mL^−1^ for 30 min. There was no difference in proliferation rates of SCs from LBWT and NBWT pigs. However, LBWT SC fusion was 15% lower (*P *≤* *0.05) without a difference in *MyoD* or *myogenin* mRNA expression in comparison with NBWT pigs, suggesting SCs are not intrinsically different between the two groups. IGF‐Ι stimulation at physiological concentrations activated downstream effectors of mTOR similarly in myotubes from LBWT and NBWT pigs. However, abundance of ribosomal protein S6 kinase 1(S6K1) was lower in myotubes of LBWT compared to their NBWT siblings (*P *≤* *0.05). These results indicate that the modest reduction in SC fusion and S6K1 expression are not the major contributors to the impaired postnatal muscle growth of LBWT pigs.

## Introduction

Low birth weight (LBWT) increases the risk of fetal mortality and neonatal morbidity, and is considered a major concern in human obstetrics and domestic animal production (Resnik 2002; Wu et al. 2006). In infants, LBWT is often associated with impaired postnatal growth and accounts for a number lifelong metabolic disorders (Barker et al. 2010; Gatford et al. 2010). Among domestic animals, pigs have the highest incidence of naturally occurring intrauterine growth restriction (IUGR), and up to 20% of newborn piglets may be considered LBWT (Wu et al. 2006). In addition, due to their similar anatomy, metabolism, and rapid postnatal growth rates, pigs have been used as a model for human infants (Bauer et al. 2003; Ferenc et al. 2014).

The neonatal period is characterized by high rates of growth compared to any other period of postnatal life, and is dominated by skeletal muscle hypertrophy (Reeds et al. 2000; Schiaffino et al. 2013). Growth of naturally occurring LBWT pigs is asymmetric, with muscles restricted the most, followed by internal organs while brain development is fully protected (Bauer et al. 2003; Rehfeldt and Kuhn 2006). Since skeletal muscles account for ~40% of the total body mass and have a crucial role in maintaining metabolic homeostasis (Yates et al. 2012), suppressed muscle development in LBWT neonates could be a major contributor to restricted postnatal growth leading to increased risk of long‐term metabolic diseases later in life (Brown 2014).

The rapid protein accretion in neonates is accompanied by an increase in the myonuclear content of muscle fibers (Davis and Fiorotto 2009). Myonuclear accretion takes place via proliferation and fusion of satellite cells (SCs), which are mononucleated stem cells with myogenic potential located under the basal lamina of myofibers (Wozniak et al. 2005). These cells provide extra nuclei into preexisting muscle fibers and contribute to fiber hypertrophy. In this regard, in rodents, the number of SCs per fiber is lower in smaller compared with larger littermates (Brown and Stickland 1993).

Proliferation and differentiation of SCs, is primarily controlled by a group of muscle‐specific transcription factors, known as myogenic regulatory factors (MRFs), including Myf5, MyoD, and myogenin (Holterman and Rudnicki 2005). The paired box transcription factor Pax7, expressed nearly ubiquitously by quiescent SCs, is coexpressed with MyoD in proliferating myoblast progeny (Brameld et al. 1998). In addition, Pax7 directs the induction of target genes, such as Myf5, which regulate SCs entry into the myogenic program (Brameld et al. 1998). By comparison, myogenin plays a key role in the processes of differentiation and formation of muscle fibers (Averous et al. 2012; Wang et al. 2012). Moreover, external stimuli like growth factors, hormones, and nutrients could also regulate proliferation and differentiation (Brown 2014).

The insulin‐like growth factor (IGF) system, which consists of multiple IGF ligands (IGF‐I and IGF‐II), IGF receptors, and IGF‐binding proteins (IGFBP), regulates myoblast proliferation, myogenic differentiation, and protein synthesis in differentiating myotubes formed from cell lines and primary myoblasts (Shen et al. 2005; Duan et al. 2010). IGFs exert their anabolic effects by activating intracellular kinase systems, including phosphoinositide 3 kinase–protein kinase B–mammalian target of rapamycin (PI3K‐PKB/Akt‐mTOR), a critical signaling cascade underlying myoblast differentiation (Halevy and Cantley 2004; Sartorelli and Fulco 2004). Activation of mTOR leads to the subsequent phosphorylation of the downstream target proteins, including eukaryotic initiation factor (eIF) 4E‐binding protein 1 (4EBP1) and ribosomal protein S6 kinase 1 (S6K1). When phosphorylated, 4EBP1 dissociates from the cap‐binding protein eIF4E, enabling it to react with eIF4G and ultimately forming the eIF4F complex, which is required for the cap‐dependent translation initiation. The activation of S6K1, through a variety of effectors, leads to an increase in mRNA biogenesis and contributes to translation initiation, which is the first and rate‐limiting step for protein synthesis (Davis and Fiorotto 2009; Ma and Blenis 2009; Laplante and Sabatini 2012).

Circulating IGF‐I concentration is 42% lower in LBWT neonatal pigs and is not affected by feeding or fasting (Davis et al. 1997). In addition, our previous data suggest that LBWT neonatal pigs have lower abundance of IGF‐I and impaired protein synthesis signaling in *longissimus dorsi* muscle (Chen et al. 2016). We hypothesized that impaired protein synthesis signaling and dysfunction of myogenic SCs contribute to the restricted skeletal muscle growth in LBWT neonatal pigs. Thus, the objectives of this study were to: (1) characterize proliferation and differentiation of SCs isolated from LBWT neonatal pigs, and (2) determine how the response to IGF‐I stimulus differs between myotubes differentiated from primary SCs of LBWT and NBWT pigs.

## Materials and Methods

### Animals and samples collection

All procedures involving animals were approved by Virginia Tech Institutional Animal Care and Use Committee. Pregnant sows had free access to water and fed daily a corn‐soybean base diet to meet NRC (2012) requirements at the Virginia Tech Swine Center. Piglets born to these sows were weighed and defined as normal birth weight (NBWT) when weight was within ±0.5 SD, or low birth weight (LBWT) when weight was ≤2 SD of the litter average. Sixteen 3‐d‐old (four pairs male and four pairs female) pigs were killed to collect the *longissimus dorsi* muscles for primary SC isolation. The NBWT and LBWT pigs were sex‐matched siblings from the same litter.

### Satellite cell isolation

**Figure 1:**
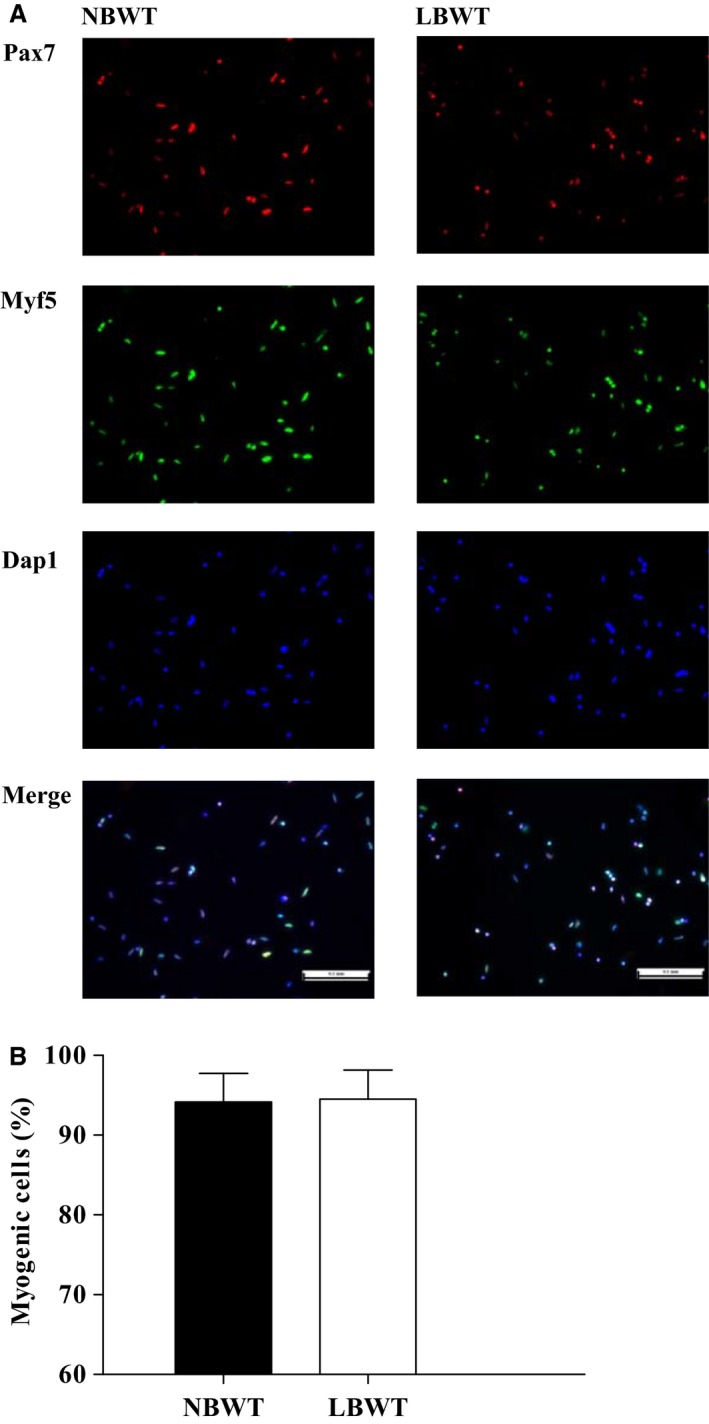
Myogenicity of original satellite cell isolates from LBWT and NBWT neonatal pigs. (A) Immunolabelling of Pax7 (*in red*) and Myf5 (*in green*) of satellite cells (Scale bar = 100 *μ*m). (B) Myogenic cells were positive for either Pax7, Myf5, or both, and expressed as the percentage of total cells. Results are means ± SE. *n *=* *8. LBWT, low birth weight; NBWT, normal birth weight.

Primary myogenic SCs were obtained from the *longissimus dorsi* muscle by protease digestion (Zhu et al. 2013). Briefly, muscles were minced after trimming off all visible fat and connective tissue, and incubated at 37°C for 1 h in a solution of 1 mg × mL^−1^ protease (Sigma, St. Louis, MO; ≥3.9 U/mg solid) dissolved in phosphate‐buffered saline (PBS). The mixture was shaken gently every 10 min during the incubation. Following enzymatic digestion, cells were separated from tissue slurry by repeated centrifugation at 400*g* for 10 min. The use of current procedure yielded SCs clean from debris and myofibrillar fragments. Myogenicity of the original culture, as measured by immunofluorescent detection of Pax7 and Myf5, was 94.2% and 94.5%, respectively, for SCs isolated from NBWT and LBWT pigs (Fig.**** 1).

### Primary cell cultures

Isolated cells were seeded in 10‐cm tissue culture dishes coated with matrigel (1/50, vol/vol; Corning, Tewksbury, MA) and grown at 37°C in growth media consisting of minimum essential medium (MEM; Gibco, Life Technologies) supplemented with 10% fetal bovine serum (FBS; Atlanta Biologicals, Lawrenceville, GA), 1% antibiotics–antimycotics (ABAM; Sigma, St. Louis, MO), and 0.1% gentamicin (Sigma) under 95% air and 5% CO_2_. When cells reached 80% confluence, they were collected using 0.25% trypsin‐EDTA (Gibco, Life Technologies). Cell mixtures were preplated for 0.5 h in noncoated culture ware to enrich for SCs, then collected and enumerated.

Cells were seeded at a density of 2 × 10^3^ cells per well in 96‐well microplates (0.34 cm^2^ per well) to assess proliferation from day 0 to day 4 following CyQUANT^®^ NF Cell Proliferation Assay Kit protocol (Invitrogen, Carlsbad, CA). Cells were also seeded at a density of 2 × 10^4^ cells × cm^−2^ in 6‐well plates (9.6 cm^2^ per well) coated with matrigel as described above and these cells were allowed to proliferate to 80% confluence in growth media, then switched to differentiation media (MEM containing 2% horse serum, 1% ABAM, and 0.1% gentamicin) to induce differentiation and myotube formation.

On day 3 of differentiation, myotubes were either washed twice with ice‐cold PBS and harvested by TRIzol^®^ Reagent (Ambion, Thermo Fisher Scientific, Waltham, MA) for RNA extraction, or myotubes were washed twice with prewarmed PBS and fasted by placing in a fresh serum‐free media for 3 h before exogenous IGF‐I administration. Myotubes were treated with human recombinant R^3^‐insulin‐like growth factor‐I (rh IGF‐I; Sigma) at 0, 25, and 50 ng × mL^−1^ for 30 min, after which protein samples were harvested by RIPA buffer (50 mmol/L Tris‐HCl, pH 7.4, 1% NP‐40, 0.25% sodium‐deoxycholate, 150 mmol/L sodium chloride, 1 mmol/L ethylenediaminetetraacetic acid [EDTA], 1 mmol/L sodium fluoride, 1 mmol/L sodium orthovanadate, and protease inhibitor [1:200, Sigma]) and frozen at −80°C for further analysis.

Immunocytochemical detection of myosin was performed to characterize SCs differentiation to primary myotubes. Myotubes were washed with ice‐cold PBS and fixed in 4% paraformaldehyde in PBS (PFA) at room temperature for 15 min followed by another PBS wash. To reduce nonspecific binding, cells were incubated in a blocking solution containing 5% horse serum (Gibco, Life Technologies) and 2% bovine serum albumin (Research Products International Corp, Mount Prospect, IL). Antimyosin (1:10 hybridoma supernatant, MF‐20, Developmental Studies Hybridoma Bank, maintained by the University of Iowa, Iowa City, IA) mouse monoclonal antibody was used as primary antibody. Cells were incubated with primary antibody in a blocking buffer overnight at 4°C and the myosin visualized using a fluorescein‐conjugated goat anti‐mouse IgG (Thermo Fisher Scientific). Cells were mounted in PBS solution containing 4, 6‐diamidino‐2‐phenylindole (DAPΙ, 1 *μ*g × mL^−1^) for the detection of nuclei. The fusion percentage was estimated from four randomly chosen fields per well, two wells per animal containing 800 nuclei per animal, and expressed as the number of DAPΙ‐stained nuclei inside myosin immunopositive myotubes divided by the total number of nuclei in the same field. Myotubes were defined as a syncytium with three or more nuclei. Myotubes size was measured from the same fields and expressed as the area (mm^2^) of myosin immunopositive myotubes. Myonuclear domain was calculated as the area of myosin immunopositive myotubes divided by the number of DAPI‐stained nuclei contained within the myotubes. A Nikon DS‐Ri1 microscope equipped with epifluroescence was used to acquire digital images. Representative images were captured and digitized with a charge‐coupled camera (SPOT, Diagnostic Imaging, Sterling Heights, MI) and NIS Elements software (Nikon).

### RNA extraction and quantitative real‐time PCR

Differentiated myotube RNA was extracted and purified following the Direct‐zol RNA Miniprep Kit protocol (ZYMO Research, Orange, CA). The concentrations of RNA were determined using NanoDrop spectrophotometer (Thermo Fisher Scientific, Wilmington, DE). RNA (200 ng × μL^−1^) was reverse transcribed into cDNA according to the manufacture's protocol of High Capacity cDNA Reverse Kit (Applied Biosystems, Foster City, CA). Real‐time qPCR was carried out with the ABI 7500 Fast Real‐time PCR cycler (Applied Biosystems). Primer sequences are presented in Table**** 1. An endogenous control gene (18 S) was used as an active reference to normalize quantification of an mRNA target. Relative mRNA expression levels were determined from the cycle threshold (Ct) values using the 2^−ΔΔCt^ comparative method.

**Table 1:** Nucleotide sequences of primers used for quantitative real‐time PCR

Gene	Direction	Primer sequence	Accession No.
*18 S*	Forward	5′‐ GTA ACC CGT TGA ACC CCA T ‐3′	AY265350
	Reverse	5′‐ CCA TCC AAT CGG TAG TAG CG ‐3′	
*IGF‐I*	Forward	5′‐ GCA CAT CAC ATC CTC TTC GC ‐3′	NM_214256.1
	Reverse	5′‐ ACC CTG TGG GCT TGT TGA AA ‐3′	
*IGF‐I receptor*	Forward	5′‐ CAT ACC AGG GCT TGT CCA AC ‐3′	NM_214172.1
	Reverse	5′‐ ATC AGC TCA AAC AGC ATG TCG ‐3′	
*Insulin receptor*	Forward	5′‐ GAA AGG GGG CAA GGG TCT AC ‐3′	XM_005654749.1
	Reverse	5′‐ CTC GGG TGC TTT GTT CTC CT ‐3′	
*IGF‐II*	Forward	5′‐ GCT CGT CTT CTT GGC CTT G ‐3′	NM_213883.2
	Reverse	5′‐ CCG GCC TGC TGA AGT AGA A ‐3′	
*IGFBP‐3*	Forward	5′‐ GTG CCT GAC TCC AAA CTC CA ‐3′	NM_001005156.1
	Reverse	5′‐ CCG TAC TTA TCC ACG CAC CA ‐3′	
*IGFBP‐5*	Forward	5′‐ AAG ATC TTC CGA CCC AAG CA ‐3′	NM_214099.1
	Reverse	5′‐ TCA CTC AAC GTT GCT GCT GT ‐3′	
*MyoD*	Forward	5′‐ ATG ACC CGT GTT TCG ACT CC ‐3′	NM_001002824.1
	Reverse	5′‐ AGG ATT TCC ACC TTG GGC AG ‐3′	
*Myogenin*	Forward	5′‐ CAG GAA CCC CAC TTC TAT G ‐3′	NM_001012406.1
	Reverse	5′‐ CTT CCT CTT ACA CAC CTT ACA ‐3′	

### Protein abundance and phosphorylation

Abundance and phosphorylation of signaling proteins were measured by western blot. Immunoblotting was performed using the following primary antibodies: *α*‐tubulin, eIF4E, IGF‐Ι receptor β, PKB/Akt (phospho‐Ser^473^), PKB/Akt and p70 S6 kinase (Cell Signaling Technology, Danvers, MA), S6K1 (phospho‐Thr^389^; Millipore, Temecula, CA), 4EBP1 (phospho‐Thr^46^; Life Technologies), 4EBP1 (Bethyl Laboratories, Montgomery, TX), eIF4E (phospho‐Ser^209^; Novus Biologicals, Littleton, CO). Myotube lysates were diluted (1:1) in a 2× sample buffer mixture (Bio‐Rad, Hercules, CA) and boiled for 5 min at 98°C. Denatured protein samples were run at the same time on triple‐wide gels (CBS Scientific, Del Mar, CA) to eliminate interassay variations. Equal amounts of total protein were loaded into each well and the samples were separated by electrophoresis. Following electrophoresis, proteins were transferred to a PVDF membrane (Thermo Scientific). Blots were blocked in 5% bovine serum albumin for 1.5 h at room temperature and then incubated with primary antibody overnight at 4°C followed by secondary antibody (Goat anti‐rabbit IgG‐HRP conjugate; Bio‐Rad) for 1 h at room temperature. Blots were developed using a chemiluminescence kit (GE Healthcare, Piscataway, NJ) for 5 min, optical density measured using a digital imager (Bio‐Rad), and densitometry analysis was performed using Image lab 4.0 (Bio‐Rad). Total protein density values were normalized to the internal loading control and the phosphorylated to total protein ratios were determined.

### Statistical analysis

Data were analyzed by PROC MIXED using SAS version 9.3 (SAS Inst. Inc., Cary, NC). For comparisons of the measurements in myoblasts and myotubes between LBWT and NBWT neonatal pigs, birth weight was the main effect, and sow and sex were the random effects. For analyses of protein abundance and phosphorylation in mTOR signaling with IGF‐Ι stimulation, birth weight and IGF‐Ι concentration were the main effects, and sow and sex were the random effects. When a significant treatment effect was detected, means were compared using Tukey–Kramer multiple comparison test. Data are expressed as the least square means ± SE and differences were considered significance at *P *≤* *0.05, unless otherwise noted.

## Results

### Primary satellite cell proliferation and differentiation

**Figure 2:**
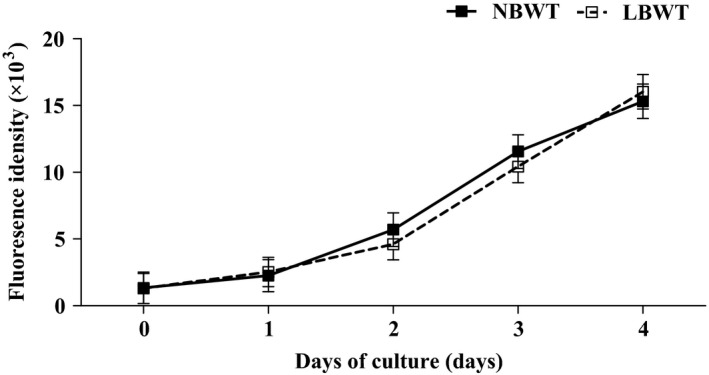
Proliferation rate of satellite cells from LBWT and NBWT neonatal piglets. Results are means ± SE. *n *=* *8. LBWT, low birth weight; NBWT, normal birth weight.

Proliferation over a 4‐d period in culture did not differ (*P *>* *0.05) between NBWT and LBWT SC isolates **(**Fig.**** 2 **)**. Eighty percent confluence was achieved after 4 days of culture in growth media in both LBWT and NBWT SC cultures.

**Figure 3:**
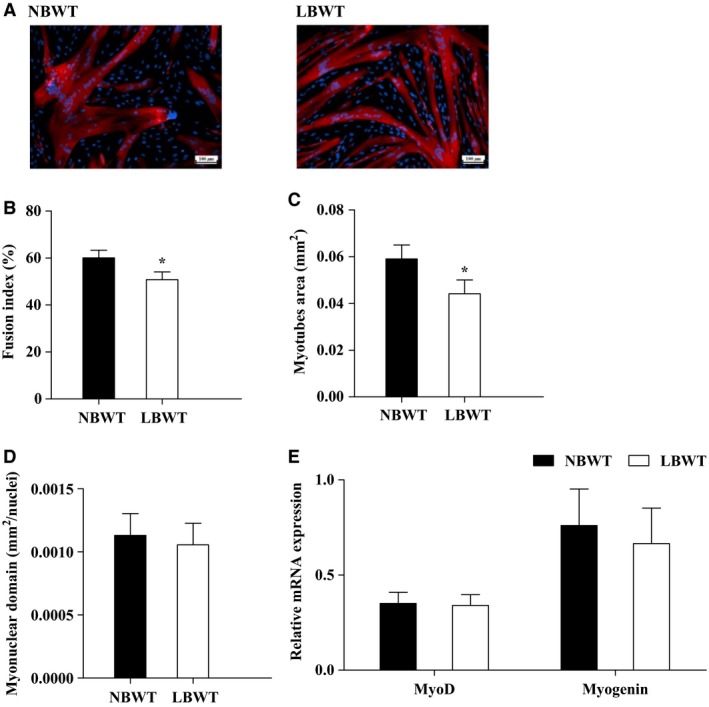
Myogenic differentiation of satellite cells from LBWT and NBWT neonatal pigs. (A) Immunolabelling in satellite cell‐derived myotubes at day 3 of differentiation (Scale bar = 100 *μ*m). (B) Fusion index expressed as the percentage of total nuclei that are located in myotubes stained in red. (C) Myotubes size expressed as the area (mm^2^) of myotubes that are myosin immunopositive (stained in red). (D) Myonuclei domain calculated as the area of myotubes (stained in red) divided by the number of DAPI‐stained nuclei inside. (E) mRNA expression of *MyoD* and *Myogenin* in myotubes of LBWT and NBWT neonatal pigs. Results are means ± SE. *n *=* *8. *P ≤* *0.05 versus NBWT pigs. LBWT, low birth weight; NBWT, normal birth weight.

As a measure of differentiation capacity, fusion indices were calculated for NBWT and LBWT SC cultures. Cultures of LBWT and NBWT SC isolates formed large myosin‐expressing structures indicating sufficient capacity for differentiation **(**Fig. 3A). The percentage of nuclei contained within myotubes was 60% and 51%, respectively, for NBWT and LWBT cultures (Fig. 3B). The modest reduction in LBWT fusion index was significantly lower (*P *≤* *0.05) in comparison with their NBWT counterparts suggesting that myotubes may be smaller. In addition, the area of myosin immunopositive myotubes was lower (*P *≤* *0.05) in LBWT than NBWT myotubes (Fig. 3C), without difference in myonuclear domain percentage between the two groups (Fig. 3D), indicating a smaller myotube size of LBWT myotubes. Expression of myogenic regulatory factors, *MyoD* and *myogenin*, did not differ in NBWT and LBWT myotubes (Fig. 3E).

### IGF system expression and function in myotubes

**Figure 4:**
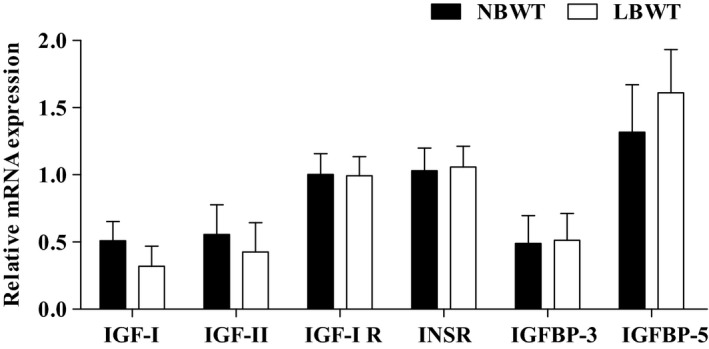
mRNA expression of IGF system in myotubes of LBWT and NBWT neonatal pigs. Results are means ± SE. *n *=* *8. LBWT, low birth weight; NBWT, normal birth weight.

**Figure 5:**
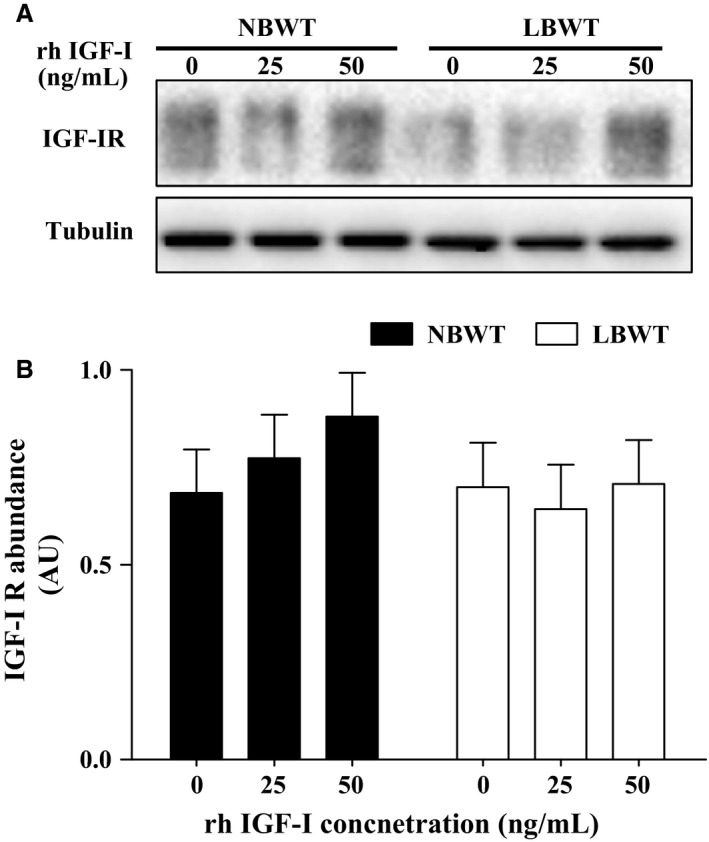
Protein abundance of IGF‐I receptor (IGF‐I R) with human recombinant R^3^‐IGF‐I (rh IGF‐I) stimulation in myotubes of LBWT and NBWT neonatal pigs. (A) Western blot image from representative samples. (B) Abundance of IGF‐I R calibrated on tubulin. Results are means ± SE. *n *=* *8. LBWT, low birth weight; NBWT, normal birth weight.

**Figure 6:**
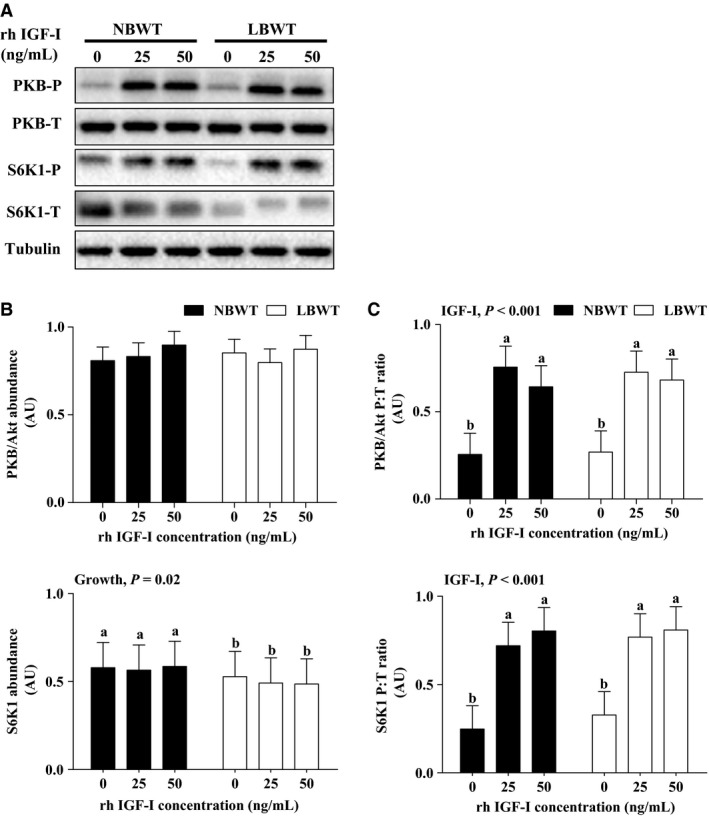
Protein abundance and phosphorylation of PKB/Akt and S6K1 in protein synthesis signaling with human recombinant R^3^‐IGF‐I (rh IGF‐I) stimulation in myotubes from LBWT and NBWT neonatal pigs. (A) Western blot images from representative samples. (B) Abundance of PKB/Akt and S6K1 calibrated on tubulin. (C) Phosphorylation of PKB/Akt and S6K1 expressed as ratio of phosphorylated to total PKB/Akt and S6K1. Results are means ± SE. *n *=* *8. Values with different letters differ significantly (*P *≤* *0.05). LBWT, low birth weight; NBWT, normal birth weight.

**Figure 7:**
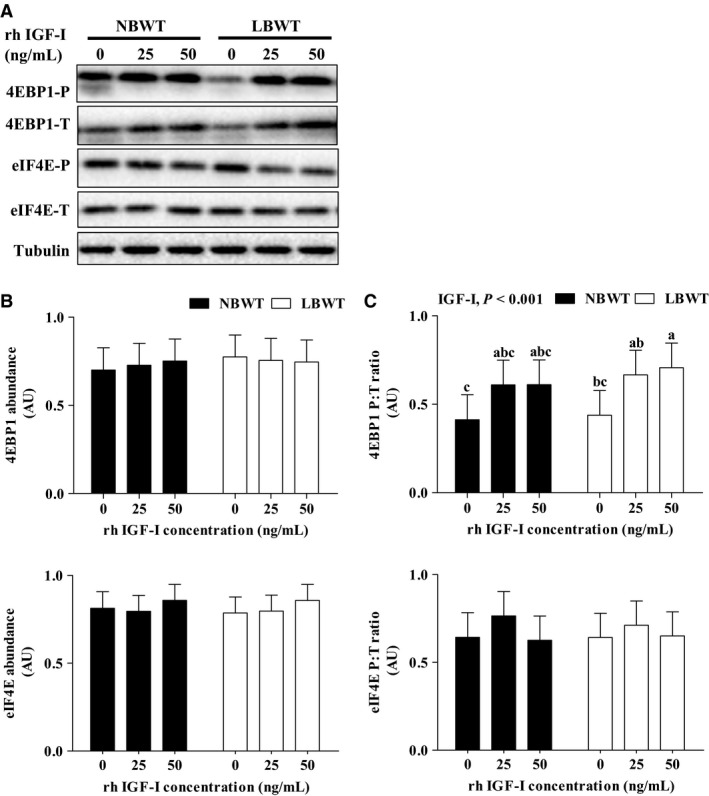
Protein abundance and phosphorylation of 4EBP1 and eIF4E in protein synthesis signaling with human recombinant R^3^‐IGF‐I (rh IGF‐I) stimulation in myotubes from LBWT and NBWT neonatal pigs. (A) Western blot images from representative samples. (B) Abundance of 4EBP1 and eIF4E calibrated on tubulin. (C) Phosphorylation of 4EBP1 and eIF4E expressed as ratio of phosphorylated to total 4EBP1 and eIF4E. Results are means ± SE. *n *=* *8. Values with different letters differ significantly (*P *≤* *0.05). LBWT, low birth weight; NBWT, normal birth weight.

Gene expression of *IGF‐Ι*,* IGF‐II*,* IGF‐Ι R*,* insulin receptor* (*INSR)*,* IGFBP‐3*, and *IGFBP‐5* was not different in myotubes from LBWT compared with NBWT neonatal pigs (Fig.**** 4). Importantly, equivalent amounts of IGF‐I receptor (IGF‐I R) were demonstrated by western blot (Fig. 5A). Stimulation of myotubes with either 25 or 50 ng × mL^−1^ human recombinant R^3^‐IGF‐I did not alter receptor protein abundance in either NBWT or LBWT myotubes (Fig. 5B). In addition, IGF‐I R effector kinase expression and phosphorylation were examined in NBWT and LBWT myotubes lysates by western blot. Following treatment for 30 min with either 25 or 50 ng × mL^−1^ rh IGF‐I, a robust increase (*P *≤* *0.05) in phosphorylation of PKB/Akt, S6K1, and 4EBP1 was noted (Figs. 6, 7). Phosphorylation patterns and relative abundance ratios for PKB/Akt, S6K1, and 4EBP1 were similar between NBWT and LBWT myotubes treated with IGF‐I irrespective of the concentration. A slight (13%) reduction (*P *≤* *0.05) in the expression of S6K1 was noted in the myotubes from LBWT compared with that from NBWT pigs. Abundance and phosphorylation of eIF4E remained unchanged after IGF‐Ι treatment in myotubes from both of LBWT and NBWT neonatal pigs (Fig. 7).

## Discussion

The current paradigm of neonatal muscle growth is that hyperplasia (increase in myofiber numbers) is completed at birth (Glass 2003; Miyazaki and Esser 2009). In this regard, muscle fiber numbers are lower in LBWT pigs compared with their NBWT littermates at birth which may limit their postnatal growth (Dwyer et al. 1994; Gondret et al. 2006; Rehfeldt and Kuhn 2006). As such, postnatal skeletal muscle growth would occur by means of enlargement and elongation of existing muscle fibers (Glass 2003; Miyazaki and Esser 2009). A major mechanism supporting muscle hypertrophy is SC‐mediated myonuclear incorporation, which precedes protein accumulation in the early stages of postnatal muscle growth (Davis and Fiorotto 2009; Ten Broek et al. 2010). A second mechanism is through an increase in protein accretion as a result of increased synthesis (Davis and Fiorotto 2009). We hypothesized that impairments in the SCs function and protein synthesis signaling contribute to the restricted skeletal muscle growth in LBWT neonatal pigs. In this study, we investigated how LBWT affects SCs proliferation and differentiation and the response to exogenous IGF‐I stimulus in myotubes differentiated from primary SCs of LBWT and their NBWT littermates, which provides novel insights into the molecular mechanisms underlying the restricted skeletal muscle growth phenotype in LBWT neonatal pigs.

In this study, SCs were isolated from *longissimus dorsi* muscle of LBWT and NBWT neonatal pigs and cultured under the same conditions to proliferate and differentiate into myotubes. There was no difference in proliferation rate of SCs between LBWT and NBWT neonatal pigs. However, fusion index of SCs into myotubes was only slightly lower with no difference in the expression of *MyoD* and *myogenin* in myotubes of LBWT compared to NBWT pigs. During postnatal growth, SCs facilitate myonuclei accretion through proliferation, followed by differentiation and fusion into existing muscle fiber in skeletal muscle in vivo (Mesires and Doumit 2002). Isolates of LBWT SCs had a slightly lower fusion index in the absence of in vivo environment, suggesting LBWT SCs may not be intrinsically different from the cells of their NBWT littermates. Thus, SC dysfunction is unlikely to be a major contributor to the impaired muscle hypertrophy observed in LBWT animals.

Myoblasts isolated from heat stress‐induced IUGR sheep fetuses proliferate at a slower rate than those isolated from animals raised in their thermoneutral zone under identical culture conditions (Yates et al. 2014). Although proliferation differed, the differentiation ability was similar between the two groups. Culturing myoblasts in sera from IUGR animals diminished myoblast proliferation rate in both IUGR and control groups indicating that extrinsic factors, for example, hormones, nutrients, may be responsible for decreased proliferation (Yates et al. 2014). It could be possible that the differences in proliferation and differentiation between sheep and pig myoblasts may be due to species or age differences or differences that resulted from the spontaneous versus experimentally induced LBWT models.

SCs express different myogenic regulatory factors at different stages of development, which drive the myogenic program (Dhawan and Rando 2005). Upon rat SC activation, transcript and protein level of myogenic regulatory factor, *MyoD*, increase within 12 h (Smith et al. 1994). In addition, proliferating SCs express *MyoD* during expansion (L'Honore et al. 2007). As SCs exit the cell cycle and undergo terminal differentiation, they continue to express *MyoD* (Gillespie et al. 2009). The onset of differentiation of SCs is marked by an increased expression of myogenic regulatory factor, *myogenin*, which promotes myotube formation (Wozniak et al. 2005). In this study, gene expression of *MyoD* and *myogenin* did not differ in myotubes between LBWT and NBWT pigs at day 3 of differentiation, in spite of lower fusion index of SCs from LBWT compared with NBWT pigs. Thus, our data suggest that the lower fusion capability is unlikely to be regulated by myogenic regulatory factors rather extrinsic factors may play a more prominent role in decreasing fusion ability in myotubes of LBWT neonatal pigs.

It is established that IGFs are potent stimulators of myogenic differentiation and hypertrophic response in skeletal muscle (Duan et al. 2010). Our previous data suggest that *IGF‐I* and *IGFBP‐5* expression are lower while *IGF‐II* and *IGF‐I R* are higher in *longissimus dorsi* muscle of LBWT compared with their NBWT littermates at birth (Chen et al. 2016). The gene expression profile of the IGF system in myotubes from LBWT and NBWT neonatal pigs has not been previously elucidated. We set out to determine whether changes in IGF system previously observed in vivo were similar in SC‐derived myotubes of LBWT and NBWT pigs. Contrary to what we expected, there were no differences in the mRNA expression of *IGF‐Ι*,* IGF‐II*,* IGF‐Ι R*,* INSR*, and *IGFBP‐3* and *‐5* between myotubes grown from SCs of LBWT and NBWT pigs. It is evident that culture environment can affect cell properties in in vitro culture systems. For instance, proliferation rate of myoblasts isolated from fetal sheep is diminished when incubated in serum from IUGR animals compared with those in regular culture medium (Yates et al. 2014). In this study, we opted to use identical culture conditions to determine whether the differences between SCs from LBWT and NBWT pigs are due to inherent factors. As such, one possible explanation for the inconsistency between the expression of IGF system in skeletal muscle in vivo and cultured myotubes may have resulted from using the same culture conditions for cells from LBWT and NBWT pigs.

In this study, a lower fusion capacity of SCs from LBWT pigs compared to NBWT pigs was not associated with altered myotube expression of *IGFs* and *myogenin* between the two groups. PI3K‐PKB/Akt‐mTOR pathway, which is a major downstream signaling of IGF‐I, mediates the stimulatory effect of IGFs on SCs differentiation and muscle hypertrophy (Halevy and Cantley 2004). Consequently, it is possible that compromised PI3K‐PKB/Akt‐mTOR pathway may explain the decreased fusion of myotubes and impaired muscle growth in LBWT neonatal pigs. To further investigate the mechanism of PI3K‐PKB/Akt‐mTOR signaling regulation in myotubes, physiological IGF‐I concentrations were administered to myotubes differentiated from myogenic SCs. IGF‐Ι stimulation increased the phosphorylation of PKB/Akt, S6K1, and 4EBP1 equally in myotubes from both LBWT and NBWT neonatal pigs, which indicated that IGF‐Ι activated downstream effectors of mTOR to a similar extent in both LBWT and NBWT pigs.

A novel finding was that protein abundance of S6K1 was significantly reduced in myotubes from LBWT than that of NBWT pigs. S6K1, a protein kinase activated by IGF‐I regulates muscle hypertrophy via mTOR signaling (Park et al. 2005). To illustrate, S6K1^−/−^ mice have lower birth weight compared with wild‐type, and the retarded growth in S6K1^−/−^ mice occurs from early embryonic (reduction of >30% at E14.5) through adult stages (reduction of ~ 15%; Klammt et al. 2008; Pende et al. 2004; Shima et al. 1998). Our previous data also suggest that protein phosphorylation of S6K1 is lower in *longissimus dorsi* muscle of LBWT compared to their NBWT littermates at birth (Chen et al. 2016). Moreover, myoblasts isolated from S6K1^−/−^ mice are smaller in size despite similar proliferation rates compared with those isolated from wild‐type animals (Ohanna et al. 2005). Although the differentiated S6K1^−/−^ myotubes have normal number of nuclei, they do not display a hypertrophic response to IGF‐I stimulation, which suggest an important role of S6K1 in fiber protein accretion (Ohanna et al. 2005). Accordingly, S6K1 is closely associated with muscle hypertrophy, and decreased protein abundance of S6K1 in SC‐derived myotubes of LBWT pigs may contribute to the reduced fusion ability of SCs and impaired postnatal muscle growth in LBWT neonatal pigs.

In conclusion, SCs from LBWT pigs do not seem to be intrinsically different compared to cells from their NBWT siblings. With IGF‐Ι stimulation, downstream effectors of mTOR were activated in a similar manner in myotubes from LBWT and NBWT pigs. However, protein abundance of S6K1 was lower in myotubes from LBWT compared with NBWT pigs, regardless of IGF‐I stimulation. Thus, the modest reduction in SC fusion and S6K1 expression are not the major contributors to the impaired postnatal muscle growth of LBWT pigs. Further studies are required to investigate how other anabolic stimuli affect SC function and activation of protein synthesis signaling in LBWT neonates.

## Conflict of Interest

The authors have no conflicts of interest, financial, or otherwise, to declare.
